# Simultaneous Multifrequency Modulated Wireless Information and Power Transfer for a Triboelectronic Monitoring System

**DOI:** 10.1002/advs.202510427

**Published:** 2025-09-28

**Authors:** Hongwei Yuan, Youngwook Chung, Ze Wang, Haojie Li, He Zhang, Sang‐Woo Kim, Keren Dai

**Affiliations:** ^1^ School of Mechanical Engineering Nanjing University of Science & Technology Nanjing 210094 China; ^2^ School of Advanced Materials Science and Engineering Sungkyunkwan University Suwon 16419 Republic of Korea; ^3^ Department of Materials Science and Engineering Yonsei University Seoul 03722 Republic of Korea

**Keywords:** information demodulation circuits, multifrequency modulation, power transmission efficiency, simultaneous wireless power and information transmission, ultrasound‐driven triboelectric nanogenerators

## Abstract

Ultrasound‐driven triboelectric nanogenerators (US‐TENGs) address energy supply challenges for implantable medical microsystems, underwater sensing and monitoring microsystems, etc., and are achieving increasingly important applications. In this work, multifrequency modulated simultaneous wireless power and information transmission (MF‐SWIPT) technology is proposed for a US‐TENG that allows both the power and information flows to be transmitted continuously without interruption. The deliberately optimized design traditionally allows complex multifrequency information demodulation circuits to consume only a few microwatts of power, making it possible to operate in the US‐TENG system. Benefiting from this new architecture, the efficiency of information transmission is increased, with almost no reduction in the power transmission efficiency. The US‐TENG‐MF‐SWIPT is demonstrated for underwater temperature monitoring microsystems and other applications; in addition to powering the microsystem for continuous operation, it is capable of transmitting information and text and further enables the modulation of microsystem operating parameters, such as alarm temperature thresholds. Therefore, US‐TENG‐MF‐SWIPT technology will greatly enhance the energy sustainability and information interaction of these microsystems.

## Graphical abstract

A ultrasound‐driven triboelectric nanogenerator multi‐frequency modulated simultaneous wireless power and information transmission (US‐TENG‐MF‐SWIPT) system is proposed for various intelligent microsystems, which increases the efficiency of information transmission multiply with almost no reduction in the power transmission efficiency. In simulation and experiments, the performance advantage of the system over single‐frequency SWIPT is verified, the application of the system in underwater monitoring microsystems is demonstrated which is able to reliably transmit energy while transmitting command information.

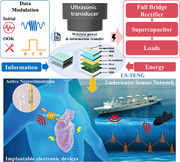

## 1 Introduction

Owing to their advantages of flexibility,^[^ ^1^, ^2^, ^3^ ^]^ ease of fabrication,^[^ ^4^, ^5^ ^]^ and low cost,^[^ ^6^, ^7^, ^8^, ^9^, ^10^ ^]^ triboelectric nanogenerators (TENGs) are rapidly being developed in many scenarios where traditional vibration generators are difficult to apply.^[^ ^11^, ^12^, ^13^, ^14^ ^]^ In particular, since ultrasound‐TENG (US‐TENG) technology has been proposed as a combinatorial solution, it has been widely used to power implantable medical microsystems^[^ ^15^, ^16^, ^17^, ^18^ ^]^ and underwater sensing/monitoring microsystems^[^ ^19^, ^20^, ^21^, ^22^ ^]^ and hybrid triboelectric–electromagnetic buoys for real‐time ocean monitoring,^[^ ^23^ ^]^ realizing long‐term energy autonomy and avoiding the inconvenience of replacing batteries. Since US‐TENG technology has been reported by scholars,^[^ ^24^ ^]^ many scholars worldwide have devoted themselves to this field, and much work has been carried out to optimize US‐TENG design from the aspects of high‐performance friction materials,^[^ ^25^, ^26^, ^27^ ^]^ device configurations and structures,^[^ ^28^, ^29^ ^]^ energy‐harvesting circuits,^[^ ^18^, ^30^ ^]^ and multifunctional triboelectric systems inspired by biological sensory perception.^[^ ^31^ ^]^ These efforts have greatly improved the energy transfer efficiency and power density of US‐TENGs. Owing to these efforts, US‐TENGs have been successfully demonstrated in pacemakers and many other important microsystems and are undergoing clinical application.^[^ ^32^, ^33^, ^34^ ^]^ While US‐TENGs have greatly contributed to sustainable energy transfer for in vivo and underwater devices, information transfer is equally essential. Through the transmission of control commands, microsystems can switch operating modes or reset parameters in real time. This capability is crucial for in vivo medical applications—such as real‐time drug delivery or adaptive neural prosthetics—which require dynamic adjustment to the patient's physiological state for more personalized and effective treatment. In underwater monitoring, it enables systems to focus more precisely on critical events, such as early seismic signals or abrupt changes in marine ecosystems, enhancing responsiveness and situational awareness.

To enable information transmission using US‐TENGs, early approaches primarily relied on integrating US‐TENGs with commercial communication modules.^[^ ^35^, ^36^ ^]^ However, such configurations led to excessive energy consumption in the communication circuitry and increased the overall size of the microsystem, significantly limiting their applicability in miniaturized or implantable platforms. In response, considerable research efforts have been directed toward integrating US‐TENGs with customized communication schemes and ultrasonic modulation techniques, particularly amplitude shift keying (ASK). For instance, one approach utilizes ultrasonic waves to excite a sensor operating on the principle of triboelectrification‐induced electroluminescence, enabling optical emission for information transfer.^[^ ^37^ ^]^ Another study introduced a micro triboelectric ultrasonic device that combines TENG and micro‐electromechanical systems (MEMS) technologies, validating the feasibility of ultrasound‐based data transmission via ASK modulation under ultrasonic excitation.^[^ ^38^ ^]^ Additionally, advanced TENG modules capable of efficient signal management have been reported,^[^ ^39^ ^]^ providing guidance for low‐energy information transmission in miniaturized microsystems. While these advances represent important progress toward self‐powered, TENG‐enabled communication, several fundamental challenges persist. Specifically, most existing TENG‐based systems with information transmission capability do not fully exploit the energy harvested by US‐TENGs and are incapable of sustaining continuous, battery‐free operation of MEMS. In addition, the influence of simultaneous information reception on energy harvesting efficiency has been largely overlooked. Consequently, the dual objectives of ensuring a stable energy supply and achieving efficient data transmission under the constrained output characteristics of TENGs remain unresolved.

Considering the above application demands, simultaneous wireless information and power transfer (SWIPT) is urgently needed for the US‐TENG. In fact, SWIPT has already had successful academic theories and industrial applications in the field of wireless communications, where information is loaded and extracted through modulation and demodulation in terms of the amplitude, frequency, phase or duty cycle of electromagnetic waves.^[^ ^40^, ^41^, ^42^, ^43^, ^44^ ^]^ The main difficulty in promoting the principles of SWIPT to US‐TENGs is the overly high energy loss in the demodulation circuitry, which is both unprofitable and unacceptable for US‐TENGs, which typically have only submilliwatt output powers. Recent studies on self‐adaptive hybrid TENG systems have demonstrated promise in real‐time environmental monitoring,^[^ ^45^ ^]^ highlighting new possibilities for extending SWIPT concepts to US‐TENG platforms. To address these limitations, our group has recently developed a low‐power demodulation circuit specifically designed for US‐TENGs,^[^ ^29^ ^]^ thereby enabling SWIPT within a unified, self‐sustaining framework. This successful breakthrough allows the US‐TENG to serve both power and information transfer functions, thus eliminating the need for additional commercial communication modules in in vivo microsystems and underwater microsystems and dramatically improving their interaction capabilities and intelligence while reducing their size and energy consumption.

Moreover, the breakthrough of US‐TENG‐SWIPT technology has opened a new research field, as its synergy optimization of energy transfer efficiency and information transfer efficiency is still an open problem. Similar transmission efficiency optimization is also a research hotspot in the field of traditional wireless communication SWIPT,^[^ ^46^, ^47^, ^48^ ^]^ in which multifrequency modulation and many other optimization mechanisms have been proposed and applied. However, the transmission efficiency optimization of the US‐TENG‐SWIPT is more difficult because the energy loss of its SWIPT circuit must be low enough to fit the output of the TENG. In this paper, through an ingenious circuit design, a multifrequency modulated SWIPT (MF‐SWIPT) technique that can be adapted to a low‐output‐power US‐TENG is proposed. Benefiting from this new architecture, the efficiency of information transmission is increased, with almost no reduction in the power transmission efficiency. In the following parts of this paper, the principle design, performance simulation, experimental testing, and functional demonstration of the US‐TENG‐MF‐SWIPT are presented in detail. Its application value will also be verified via an underwater temperature monitoring microsystem case.

## 2 Results and Discussion

### 2.1 System Architecture and Workflow

**Figure 1:**
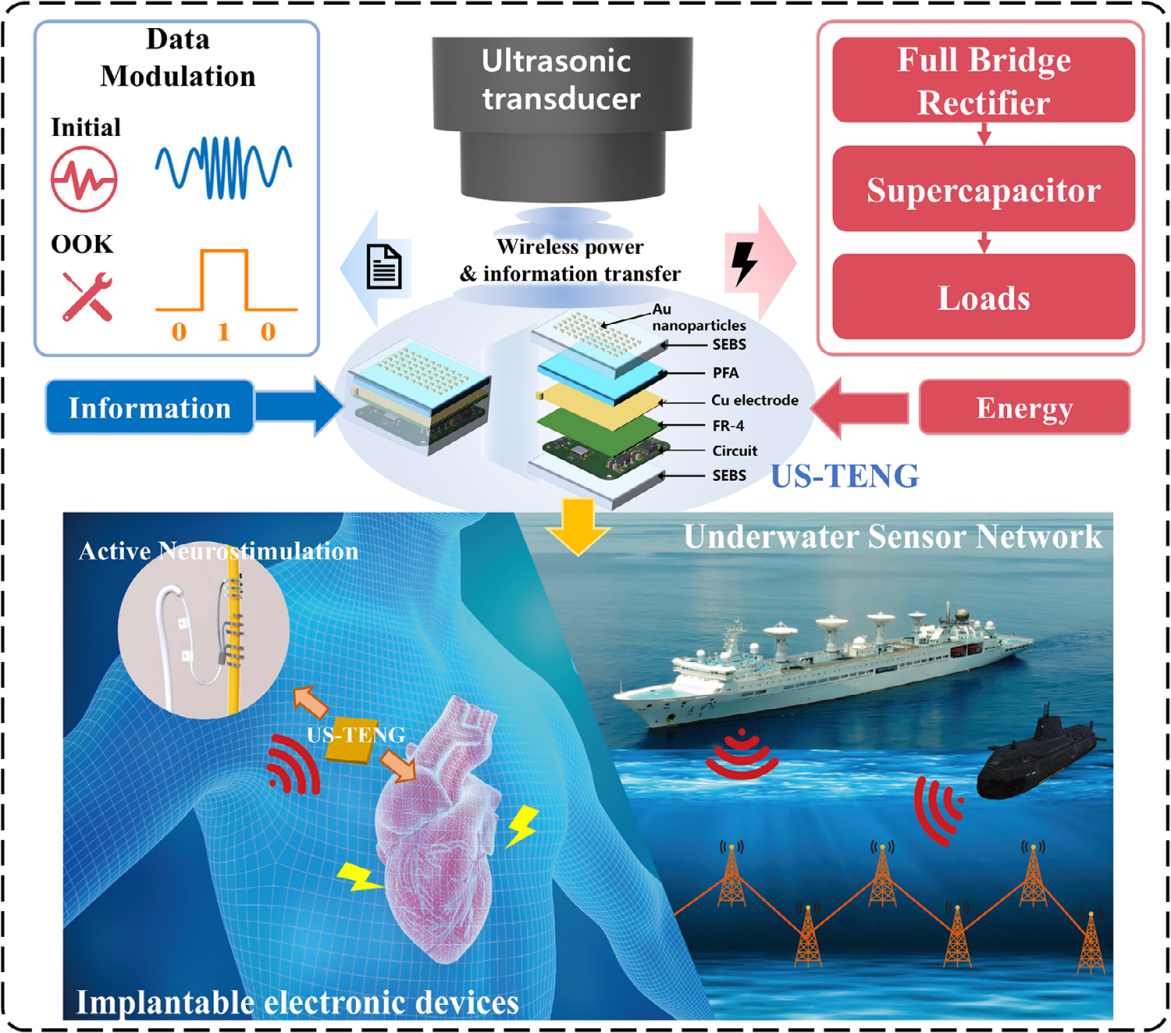
A workflow diagram showing the proposed multifrequency information demodulation and simultaneous power transmission system based on US‐TENGs and their application.

US‐TENG‐MF‐SWIPT technology aims to provide sustainable and robust integrated solutions of energy supply and information interaction for in vivo medical microsystems and underwater monitoring microsystems, as shown in **Figure** **1**. The architecture of the entire microsystem consists of three components: the waterproof packaged TENG device, the MF‐SWIPT integrated circuits, and the functionalized load module.

The US‐TENG presented in this study adopts a structure similar to that reported in.^[^ ^5^ ^]^ The device is designed in a dual‐electrode contact‐separation configuration, where the electrodes are composites with gold nanoparticles mixed in a styrene‐ethylene‐butylene‐styrene (SEBS) layer, and the friction layer material is perfluoroalkoxy, as shown in Figure S2 (Supporting Information). The entire device is integrally waterproof packaged with SEBS material and thus is capable of operating in wet liquid environments, either in vivo or underwater. Previous research has demonstrated that this special TENG device can provide hundreds of microwatts of power output under US excitation over a wide frequency range, thus making it possible to realize MF modulation.

Via digitized multifrequency modulation at the US excitation side, after ultrasound propagates through the human body or a water body to the TENG device, it can generate variable‐frequency currents with information loaded. The current generated by the TENG device is then input to the integrated MF‐SWIPT circuit. After passing through the rectifier bridge, most of the power is stored in a supercapacitor and further serves as a reliable power source for the functionalized load module; only a small amount of power passes into the information demodulation circuit, where bit quantized digital information can be obtained and further serves as a control command for the functionalized load module. Because the ultrasound and TENG electrical signals can be switched between many different frequencies, the information transmission of this SWIPT can be performed in a multibit quantized manner, which is an important reason for its high transmission efficiency. Via digitized multifrequency modulation at the US excitation side, after ultrasound propagates through the human body or a water body to the TENG device, it can generate variable‐frequency currents with loaded information. The current generated by the TENG device is then input to the integrated MF‐SWIPT circuit. After passing through the rectifier bridge, most of the power is stored in a supercapacitor and further serves as a reliable power source for the functionalized load module; only a small amount of power passes into the information demodulation circuit, where bit quantized digital information can be obtained and further serves as a control command for the functionalized load module. Because the ultrasound and TENG electrical signals can be switched between many different frequencies, the information transmission of this SWIPT can be performed in a multibit quantized manner, which is an important reason for its high transmission efficiency.

With the power‒information dual drive of the MF‐SWIPT integrated circuits, functionalized load modules with multiple sensors, processors, and actuators can be operated intelligently and autonomously over the long term. For example, the microsystem used for in vivo electrical stimulation therapy can change the intensity, frequency and other working parameters of electrical stimulation according to the command information transmitted by the TENG‐SWIPT system to obtain better therapeutic effects; moreover, the underwater monitoring microsystem used for detecting temperature, oxygen content and other environmental factors can change its sensor operation mode and alarm threshold according to the command information transmitted by the TENG‐SWIPT system to obtain better monitoring effects. Notably, such information transmission and intelligent operation are performed completely by TENG devices and SWIPT circuits without adding any additional communication devices or modules, which is a fascinating aspect of this technology.

### 2.2 Circuit Topology Optimization and Simulation

**Figure 2:**
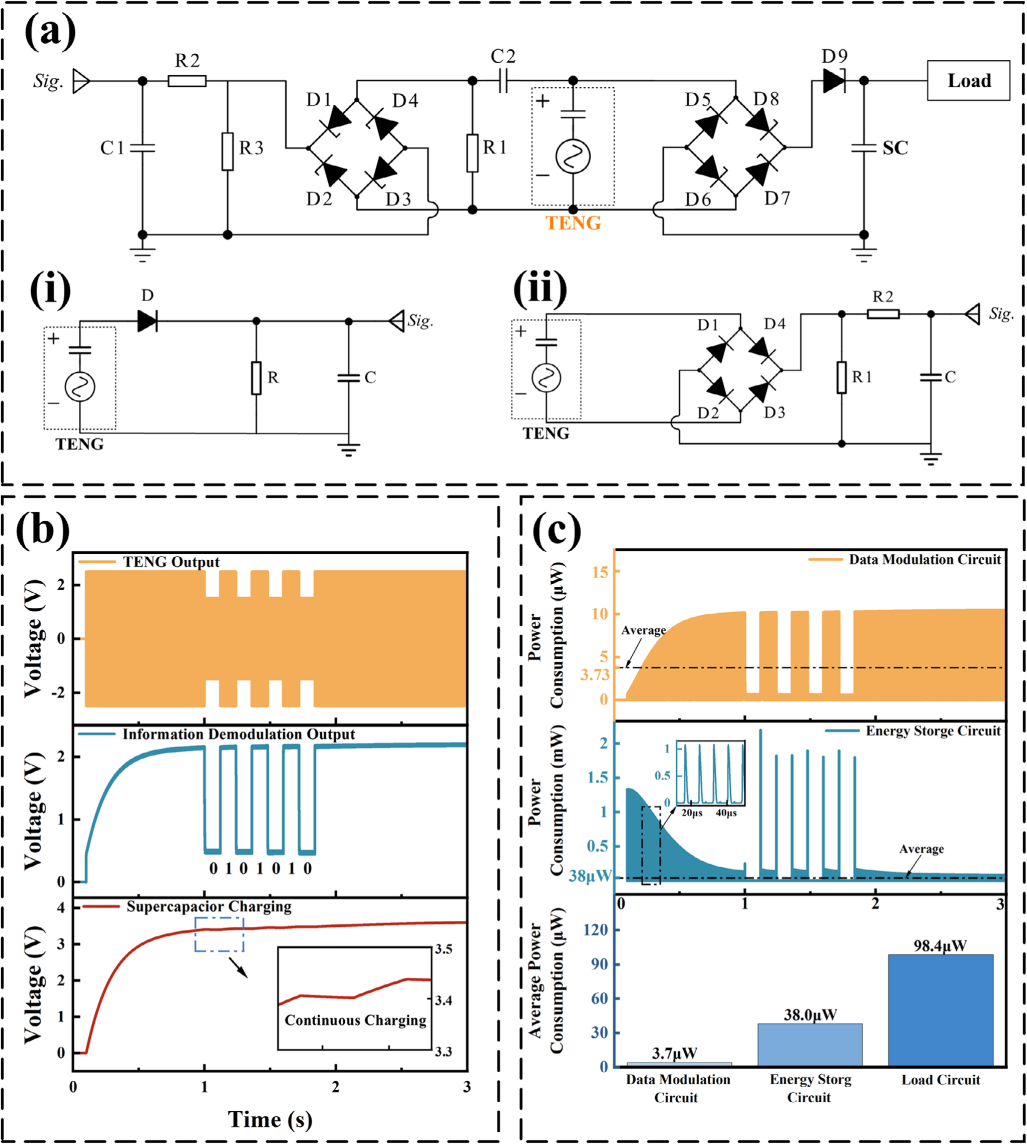
a) The proposed circuit for multifrequency information demodulation and a simultaneous energy transmission system (i: traditional information demodulation circuit; ii: circuit proposed in previous work). b) Simulation results of simultaneous energy and information transmission of the circuit proposed in this work. c) Power consumption comparison between the main components of the proposed circuit.

To realize highly efficient simultaneous wireless power and information transmission at extremely low power costs, a novel US‐TENG‐MF‐SWIPT circuit design is proposed, as shown in **Figure** **2**a. Clearly, it is an evolutionary upgrade of our previous work shown in (ii) and is fundamentally different from the conventional information demodulation circuit shown in (i). In the proposed MF‐SWIPT circuit, R1 and C1 form a high‐pass filter circuit, whereas R2, R3, and C2 form an information demodulation circuit. Therefore, the optimal design of the circuit mainly depends on the settings of the four parameters R1, R2, R3, C1, and C2. To achieve simultaneous optimization of both energy and information aspects, an optimization model is developed, as shown below.

The output signal of high‐pass filtering is directly related to its cutoff frequency, which is determined by R1 and C1:

(1) $$f_{0}=\frac{1}{2\pi R_{1}C_{1}}$$

Specifically, the output signal of the high‐pass filter circuit obeys the following decay law:

(2) Uhout=121+f0f0ff2Uin

where, *U_hout_* represents the output voltage of the demodulation circuit; *U_in_* represents the input voltage of the high‐pass filter circuit; and f is the frequency of the TENG output signal (25 kHz, 50 kHz, 80 kHz, and 125 kHz in this work).

For information demodulation circuits, the charge/discharge time constant (*t_ch_* = *xx*,*t_dch_* = *zz*) is its most important metric, which determines whether the rising and falling edges of the demodulated signal are sharp. If the signal is more closely aligned with a standard square wave, the information in the signal is more likely to be detected correctly.

In terms of energy, the vast majority of the power consumption of the high‐pass filter circuit and the information demodulation circuit is generated by resistors and capacitors, and the nonideal power consumption generated by other components, such as diodes, is negligible in comparison. It can be expressed as follows:

(3) $$P_{ed}=\frac{1}{t_{d}}\int_{t_{1}}^{t_{2}}\left({\left(U_{in}\right)}^{2}/Z_{hp}+{\left(U_{hout}\right)}^{2}/Z_{dm}\right)dt$$

where, *P_ed_* represents the average power consumption of the high‐pass filter circuit and the information demodulation circuit in one information cycle; $Z_{hp}=xx$ represents the impedance of the high‐pass filter circuit; $Z_{dm}=xx$ represents the impedance of the information demodulation circuit, where // represents impedance parallel calculation, A//B=1/1(1/1AA+1/1BB)(1/1AA+1/1BB); *xx* represents the start and stop time of the information bit; and *t_d_* represents the duration of one information bit.

(4) $$\begin{array}{c} \min_{\left\{R1,R2,R3,C1,C2\right\}}\frac{1}{x}\sum_{i=1}^{x}P_{ed}^{i}\\ s.t.\min\left\{\Delta U1,\Delta U2,\text{…},\Delta U_{N-1}\right\}>V_{dmin}\\ \max\left\{\Delta U1,\Delta U2,\text{…},\Delta U_{N-1}\right\}<V_{s}/\left(N-1\right)\\ t_{dch}<\alpha t_{d}\mathrm{and}t_{ch}<\beta t_{d}\\ t_{ch}>\gamma \cdot 1/f_{\min}\mathrm{and}t_{dch}>5\times 1/2f_{\max}\\ U_{N}<V\mathrm{and}U_{0}>0 \end{array}$$

where *x* is the number of symbols required to transmit information, which satisfies *N^x^*‐1≥*M* and *N^x‐^* ^1^‐1<*M*; *M* is the decimal value corresponding to the transmitted information; *N* is the number of frequencies used in the system; Δ*U*1, Δ*U*2, …, Δ*U*_*N* − 1_ is the difference in the demodulated voltages corresponding to adjacent frequencies; *V_dmin_* is the minimum recognizable voltage; *V_s_* is the supply voltage of the microprocessor used to demodulate information; α and β are the message period coefficients; and *f*_min_ and *f*_max_ are the minimum and maximum frequencies of the TENG output signal, respectively.

The first two constraints are bit voltage resolution ability constraints that ensure the reliable identification of information. The third constraint is the bit temporal resolution ability, which ensures that the information conversion time is short enough to ensure that the duration of the demodulated information is equal to the set information duration. The fourth constraint is the voltage ripple per bit time constraint, which ensures that the data within the unit bit time remain unchanged.

Compared with the conventional configuration, a significant change in the proposed circuit configuration is the replacement of the half‐bridge rectifier by the full‐bridge rectifier, and this change ensures that the optimization problem mentioned above is solvable. Otherwise, for the conventional circuit configuration, the feasible domain of the constraints in the optimization problem is unsolvable under the low‐power TENG, which means that it is impossible to achieve reliable information extraction. Owing to the increase in the ultrasound frequency, it can use fewer bits to represent the same information. On the one hand, it reduces the time of information transmission, and on the other hand, the power consumed by the information demodulation circuit is also greatly reduced.

Based on the above optimization model, the optimal design of the capacitor and resistor parameters in MF‐SWIPT is carried out (Note S1, Supporting Information). The feasible domain of the cutoff frequency is first determined. The information loaded by the multifrequency excitation can be demodulated and extracted by the Microcontroller Unit if and only if the cutoff frequency is within this feasible domain. The cutoff frequency is negatively correlated with the resistance of the filter circuit, which means that it is also negatively correlated with the power consumption of the circuit. Therefore, to achieve a lower power consumption of the circuit, the cutoff frequency in this paper is set to the upper bound of its feasible domain, which is 63 kHz. To achieve a cutoff frequency of 63 kHz, while considering the package size and versatility of commercial capacitors and resistors, the filter circuit is set to R1 = 250 KΩ and C1 = 10 pF. For the information demodulation circuit, the single message transmission time in this paper is set to 120 ms. In this case, considering the requirements of Constraint 4 for the charging and discharging time constants, R2 = 5.1 MΩ and C2 = 0.047 nF are set. In addition, the current limiting resistor R3 is set to 10 MΩ to further reduce the power consumption of the circuit.

The BER performance of the proposed circuit under different SNR is analyzed through theoretical calculations and simulations (Note S2, Supporting Information). The results indicate that the BER decreases with increasing SNR, and the simulated values gradually approach the theoretical predictions. At low SNR, symbol errors are more frequent due to noise‐induced misidentification, whereas at higher SNR the received symbols match the ideal levels. These results demonstrate that the information loading method reliably transmits multiple bits per symbol with high fidelity under varying noise conditions.

For the optimized MF‐SWIPT circuit, its energy and information transfer performance for the US‐TENG is first simulated via LTspice software. In the simulation, in the first stage, the TENG is excited with high‐frequency ultrasound at 125 kHz to charge the energy storage capacitor as fast as possible, and in the second stage, the TENG is alternately excited with modulated ultrasound at 125 kHz/25 kHz to validate the simultaneous transmission of power and information of the MF‐SWIPT system. As shown in Figure 2b, the TENG has a higher voltage output of more than 2 V at 125 kHz, whereas its output is between 0 V and 1 V at 25 kHz. In terms of information transmission, the output of the information demodulation circuit in the second stage has the characteristic of regular alternating high and low levels, enabling it to be easily converted to 0 or 1 information. In terms of energy transfer, the voltage of the storage capacitor increases continuously in both the first and second phases, indicating that energy transfer is not interrupted by information transfer. In other words, this TENG‐based electronic system truly realizes parallel and noninterference of energy transfer and information transfer in the time domain.

Furthermore, the energy consumption performance of the US‐TENG‐MF‐SWIPT is analyzed via simulation, as shown in Figure 2c. Quite naturally, the energy consumption of each of the information demodulation circuits and the charging circuit varies in the AC at the same frequency as the ultrasound and TENG outputs. However, the peak value of the power consumption of the information demodulation circuit gradually increases as the energy storage capacitor is charged more fully, whereas the opposite is true for the power consumption of the charging circuit; this is because the dominant power consumption of the information demodulation circuit is determined by the filter resistor R1, and the voltage across its terminals gradually increases as the filter capacitor C1 is charged fully. The dominant power consumption of the charging circuit is determined by the diodes, and the voltage drop across them decreases as the storage capacitor is charged fully. Overall, the average power consumption of the information demodulation circuit and the charging current is very low, at only 3.7 µW and 38.0 µW, respectively. The majority of the power output from the TENG, which is as high as 98.4 µW, is utilized to power the loads. This strongly proves that the US‐TENG‐MF‐SWIPT circuit is energy efficient and of practical value.

**Figure 3:**
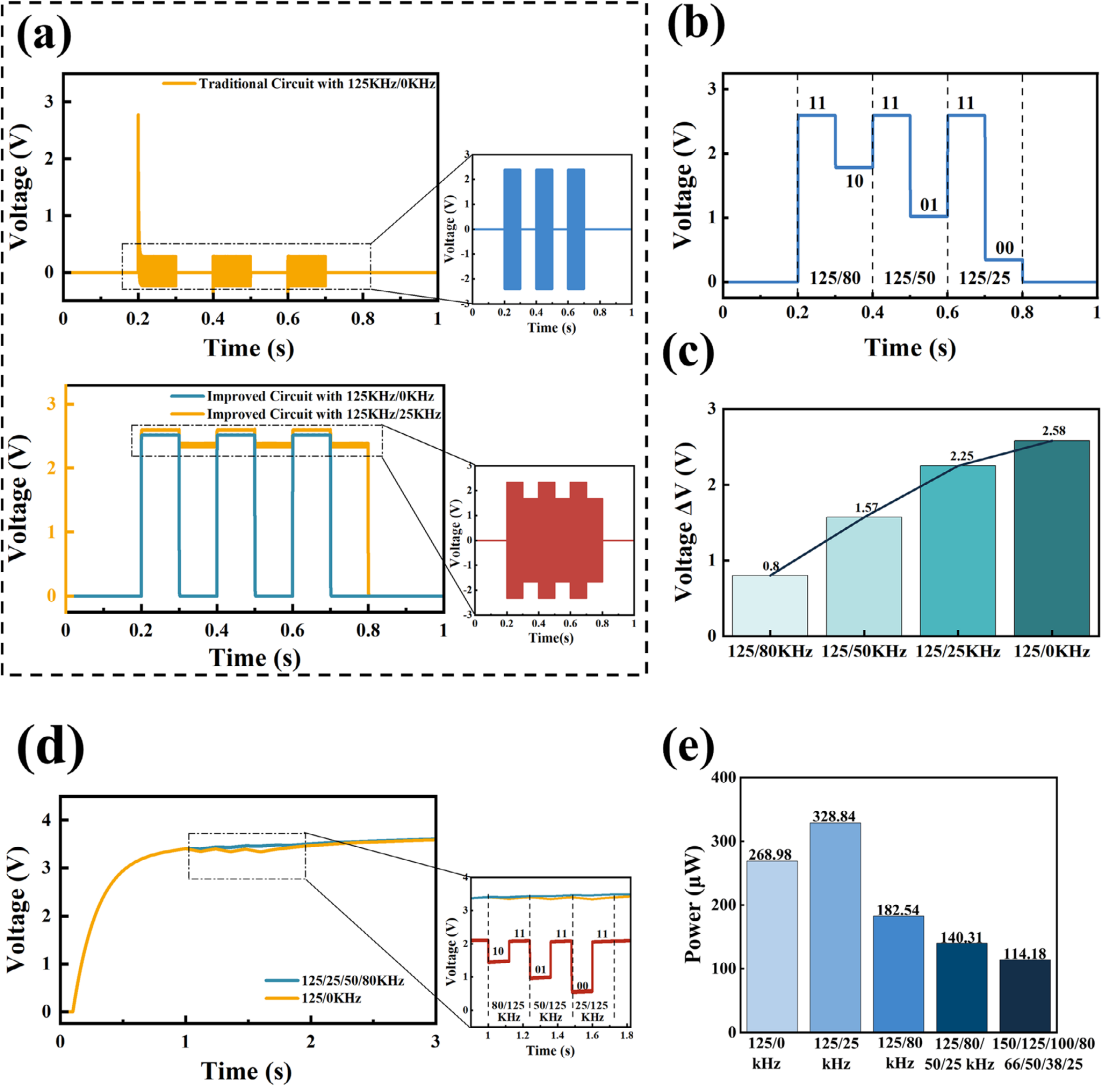
a) Comparison of the information demodulation processes between the traditional information demodulation circuit and the circuit proposed in previous work. b) Simulation results of information demodulation at different frequency TENG outputs. c) Difference in the output voltages of the proposed information demodulation circuits with different frequency combinations of TENG outputs. d) Comparison of the storge capacitive voltage simulation results in multifrequency switching mode and single‐frequency switching mode. e) Consumption power of the demodulation circuit with different frequency combinations for the same information.

To clearly demonstrate the innovations and advantages of the proposed MF‐SWIPT, a simulation analysis of the information demodulation performance with two comparison circuits is carried out, as shown in **Figure** **3**a. For the conventional information demodulation circuit shown in Figure 2ai, even with the single‐frequency operation mode of 125 kHz/0 kHz with discontinuous energy transfer (0 kHz represents no ultrasonic excitation input), it is completely unable to demodulate the existence and nonexistence of the TENG signal into high‐ and low‐level outputs; this is because the traditional information demodulation circuit lacks full‐bridge rectification, resulting in an unsolvable feasible domain determined by constraints (1)–(4) in the SWIPT system optimization problem model. Specific proofs and analyses are given in Note S1 of the Supporting Information. The US‐TENG‐SWIPT circuit proposed in our previous work addresses this critical shortcoming and thus is enabled to output high‐level and low‐level demodulated signals correctly in 125 kHz/0 kHz single‐frequency operation mode. However, owing to the lack of a filtering circuit, it is still unable to extract different frequency information in 125 kHz/25 kHz multifrequency operation mode and erroneously outputs high levels all the time. However, owing to the lack of a filtering circuit, it is still unable to extract different frequency information in 125 kHz/25 kHz multifrequency operation mode and erroneously outputs high levels all the time.

In fact, the 125 kHz/25 kHz dual‐frequency operating mode above is just one case of our US‐TENG‐MF‐SWIPT system, which is far from reaching the upper limit of its performance. As the variety of modulation frequencies increases, the information transmission rate of the MF‐SWIPT system increases in response. In Figure 3b, the information demodulation performance of the MF‐SWIPT system in the four‐frequency operation modes of 125 kHz/80 kHz/50 kHz/25 kHz is demonstrated. The output of the demodulation circuit at the four modulation frequencies is four different levels: 125 KHz corresponds to a 2.58 V output, representing the 11 information; 80 KHz corresponds to a 1.78 V output, representing the 10 information; 50 KHz corresponds to a 1.01 V output, representing the 01 information; and 25 KHz corresponds to a 0.33 V output, representing the 00 information. The level outputs at these different modulation frequencies have large enough gaps (as shown in Figure 3c) to be identified by the microcontroller as different digital information. Considering the attenuation of ultrasonic energy transmission during operational scenarios, which impacts the accuracy of information demodulation, the minimum detectable signal level difference is currently measured at 680 mV. After propagation through aqueous media, this value attenuates to 679.943 mV (99.992%), while transmission through biological tissues further reduces it to 674.175 mV (99.143%). Critically, the attenuated signals remain within the normal recognition threshold of the microprocessor, thereby ensuring sustained system functionality without operational compromise. As a result, the quarter‐frequency operation mode can significantly increase the information transmission rate of the US‐TENG‐MF‐SWIPT. Moreover, the charging curve of the storage capacitor is shown in Figure 3d indicates that the energy transmission in the quad‐frequency operation mode can still be continuously maintained. To verify the continuous demodulation capability, simulations covering all 12 possible symbol transitions in the four‐frequency mode were performed (Figure S8, Supporting Information). Each transition was successfully recognized and demodulated, confirming the system's ability to transmit multiple symbol groups continuously.

To comprehensively evaluate the performance advancement of MF‐SWIPT in terms of both energy transfer and information transfer, the average charging powers in the single‐frequency operation model, dual‐frequency operation mode, and quad‐frequency operation mode are shown in Figure 3e. The information represented in the simulation is the lowercase letter *x* (the corresponding ASCII number is 122), and the numbers of bits required to represent it in binary, ternary, quaternary, and octal are 7, 5, 4, and 3, respectively. To ensure the power of energy storage, a combination with a higher frequency is preferred. While maintaining the same information rate as the single‐frequency operation mode, the dual‐frequency operation mode provides a significant improvement in energy transfer performance, with a power consumption of 328.84 µW in the dual‐frequency operation mode of 125 kHz/80 kHz, which is an increase of 22.25%. While doubling the information transfer rate, the 125 kHz/80 kHz/50 kHz/25 kHz quad‐frequency operation mode still has a power consumption of 140.31 µW, corresponding to a 47.84% decrease compared with that of the single‐frequency operation mode. Furthermore, by extending the scheme to an octal‐frequency operation mode (150/125/100/80/66/50/38/25 kHz), the corresponding power consumption is 114.18 µW, representing an additional 18.62% reduction compared with the quaternary case. These findings strongly indicate that MF‐SWIPT has a significant improvement in comprehensive performance over the single‐frequency operation mode and that the upper limit of its performance still has great potential to be an open problem.

### 2.3 Experimental Test and Application Demonstration

**Figure 4:**
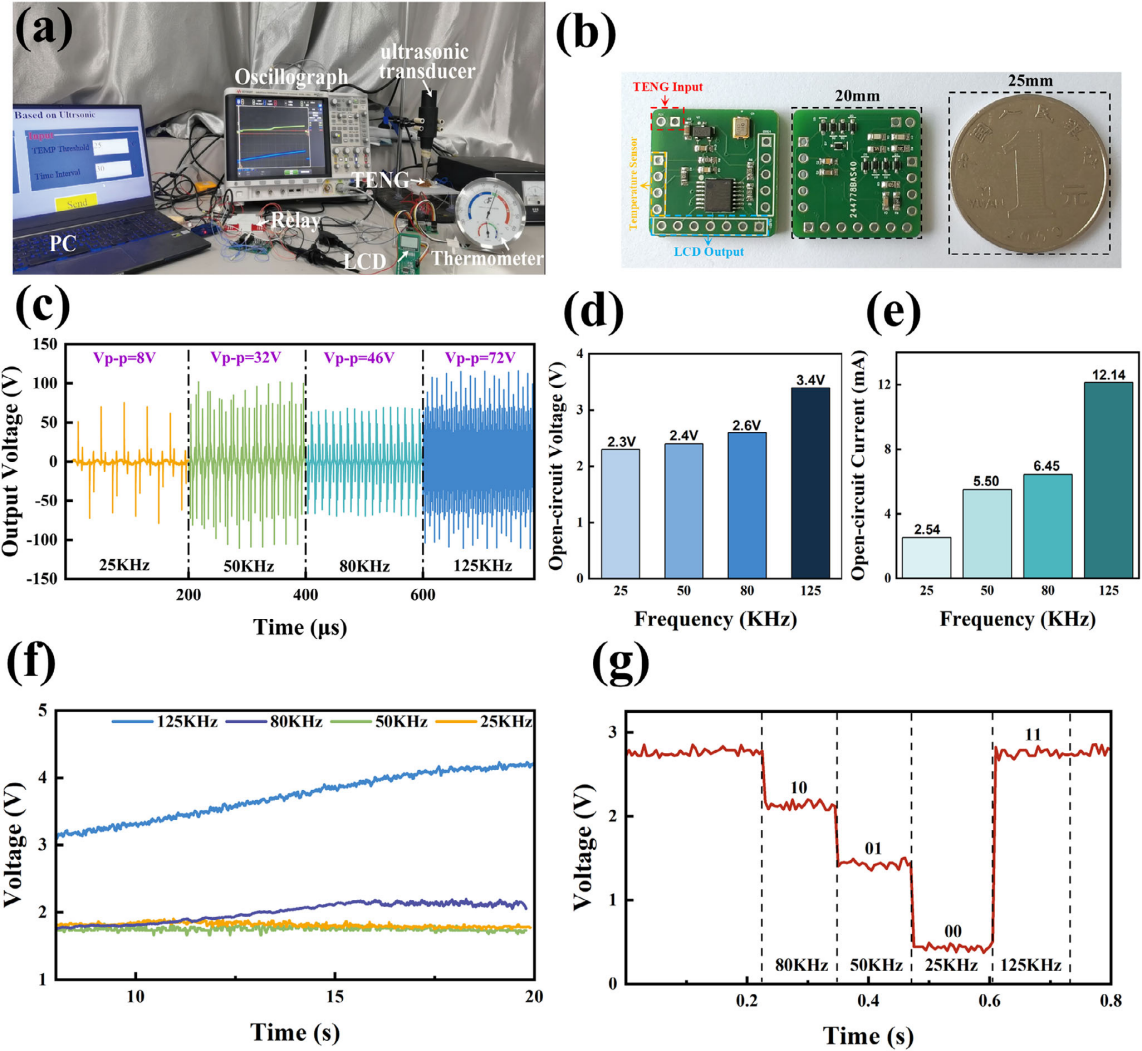
US‐TENG‐MF‐SWIPT experimental test. a) Experimental setup for multifrequency information demodulation and simultaneous energy transmission system using the US‐TENG. b) Comparison of the proposed circuit with a coin worth one yuan. c) US‐TENG output at different ultrasound frequencies. d) Short‐circuit voltage at ultrasound frequencies from 25 kHz to 125 kHz. e) Short‐circuit current at ultrasound frequencies from 25 kHz to 125 kHz. f) Storage capacitor voltage at ultrasound frequencies ranging from 25 kHz to 125 kHz. g) Experimental results of information demodulation at ultrasound frequencies from 25 kHz to 125 kHz.

Based on the above MF‐SWIPT circuit design, a prototype of the electronic system is fabricated, and a demonstration system of US‐TENG‐MF‐SWIPT is constructed, as shown in **Figure** **4**a. In this system, multiple signal generators of different frequencies are connected to the ultrasonic transducer through a single programmable relay. The information modulation module controls the relay to dynamically switch between different signal sources, thus generating an ultrasound with dynamically modulated frequencies. Driven by variable‐frequency ultrasound, the TENG generates an electric current, which is then processed by the MF‐SWIPT circuit and directed to the energy output and the information output. Finally, the energy output and the information output drive thermometers and other loads together to realize specific functions. Remarkably, all the MF‐SWIPT circuits are integrated on a double‐sided PCB with a size of only 20 mm×20 mm (Figure 4b; Figure S5, Supporting Information). Therefore, it can be conveniently stacked and integrated with microscale TENG devices for applications in size‐constrained scenarios, such as implantable medical systems and underwater monitoring nodes.

Specifically, the outputs of the TENG under ultrasonic driving at different frequencies of 25 kHz, 50 kHz, 80 kHz, and 125 kHz are shown in Figure 4c, and its corresponding peak to peak value ranges from 8 V to more than 70 V. The data show that the output voltage increases with frequency, which can be attributed to accelerated charge transfer during rapid contact–separation cycles, together with enhanced acoustic–mechanical coupling. The rising trend also suggests that the mechanical resonance frequency of the TENG structure has not yet been reached within the tested frequency range. After the TENG output is input into the charging circuit, its voltage RMS and current RMS are shown in Figure 4d,e, respectively. Both of them increase with increasing ultrasound frequency, and the trend is especially pronounced for the current RMS. As a result, as shown in Figure 4f, the TENG can realize charging of the storage capacitor under ultrasonic driving at all different frequencies, and when the frequency is higher, the maximum charging voltage of the storage capacitor is also higher. In terms of information transfer, the output signals of the TENG under four different frequencies of ultrasonic excitation can be demodulated into four different levels, namely, 125 kHz, 80 kHz, 50 kHz, and 25 kHz, corresponding to 2.596 V, 2.158 V, 1.605 V, and 0.831 V, respectively, which can represent four different types of digital information, namely, 11, 10, 01, and 00. These experimental results validate the previous analysis of the performance advantages of MF‐SWIPT in the previous simulation section.

**Figure 5:**
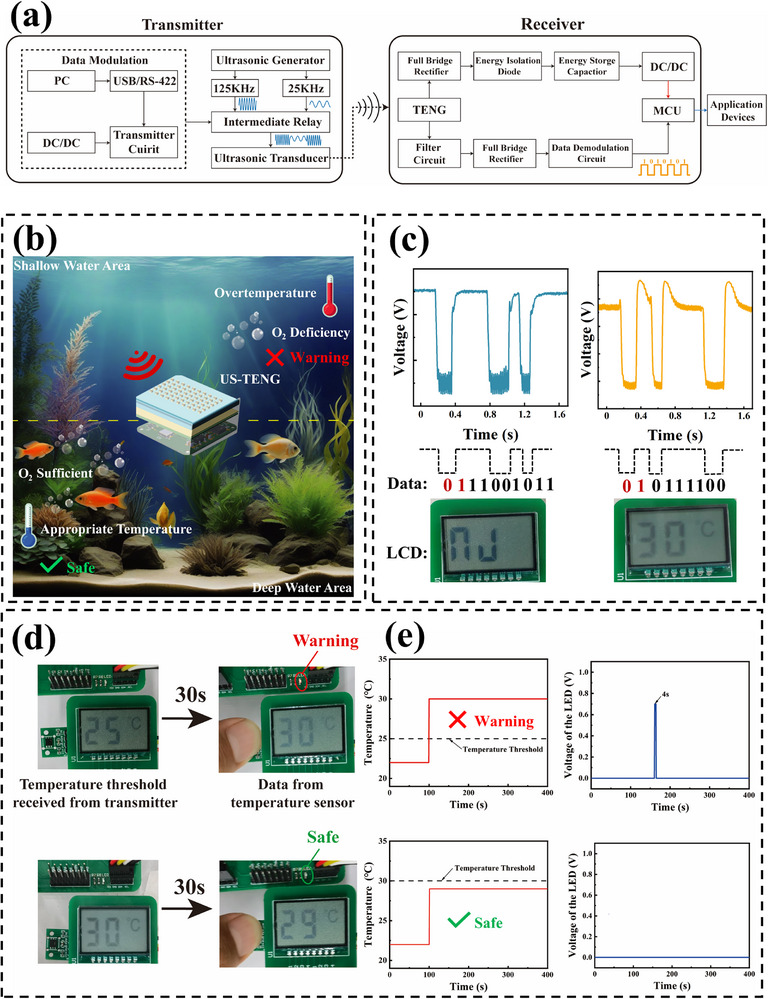
A workflow diagram for US‐TENG‐MF‐SWIPT and its application in underwater temperature monitoring microsystems a) A workflow diagram for a multifrequency information demodulation and simultaneous energy transmission system. b) Multifrequency information demodulation and simultaneous energy transmission system based on the US‐TENG for fish pond underwater temperature monitoring. c) Operation of the system that transmits alphanumeric and numeric information. d) Operation of the system that monitors the temperature and alarms.

Finally, the practical value of US‐TENG‐MF‐SWIPT is demonstrated through an application scenario of underwater temperature monitoring. In this scenario, the US‐TENG‐MF‐ SWIPT system receives the ultrasound energy and command information sent by the base station and then powers and controls the temperature sensors to operate in the appropriate mode; thus, it can send a warning alert when the surrounding temperature is anomalous. Specifically, text or other common forms of commands can be transmitted in the form of digital information by encoding (**Figure** **5**a,b). The voltage output of TENG at 25 kHz and 125 kHz is shown in Figure S6 (Supporting Information). Figure 5c shows the process and performance of transmitting text content, such as “nJ” and “30”. From top to bottom, they are the TENG output voltage signal, the demodulated digital information, and the corresponding text content. In Figure 5d and e, the powering and controlling performance of the temperature monitoring node is demonstrated. In Figure 5d, the temperature threshold of the temperature monitoring node is first set to 25 degrees Celsius through the information transmission of US‐TENG‐MF‐SWIPT. Afterward, when the surrounding temperature rises above the threshold, the temperature sensor reliably senses this change and realizes the alarm under the power of the TENG and energy storage capacitor. In Figure 5e, the temperature threshold is first changed from 25 degrees Celsius to 30 degrees Celsius through information transmission via the US‐TENG‐MF‐SWIPT system. The calculated operational lifetime under continuous monitoring is 17 hours (Note S3, Supporting Information), which is sufficient to preliminarily meet the application requirements. When the ambient temperature is 29 degrees Celsius, the temperature sensor can accurately determine that the state at this time is lower than the threshold and will not trigger an alarm. The detailed process is shown in Movie S1 (Supporting Information). These results show that US‐TENG‐MF‐SWIPT can not only reliably power the autonomous microsystem but also conveniently and in real time adjust the working mode and working parameters of the microsystem to best meet the current application needs.

## 3 Conclusion

This paper proposes a US‐TENG‐MF‐SWIPT system for implantable medical microsystems, underwater monitoring microsystems, etc., which is capable of simultaneous energy and information transfer at high rates. From the modeling perspective, the theoretical analysis proves that the design of full‐bridge rectification and high‐pass filtering is the key trick for MF‐SWIPT to achieve both energy and information transmission without mutual interference at low‐power TENG inputs. From the simulation perspective, the performance advantage of US‐TENG‐MF‐SWIPT over single‐frequency SWIPT is verified, which can increase the energy transfer rate by 47.35% to 140.31 µW while keeping the information transfer rate unchanged or double the information transfer rate with almost no decrease in the energy transfer rate. Compared with conventional wireless modules consuming ≥2.6 mW, the proposed system achieves ultra‐low power of 140.31 µW, fully compatible with TENG output (Note S4, Supporting Information). Finally, from an experimental perspective, the application of US‐TENG‐MF‐SWIPT in underwater monitoring microsystems is demonstrated, which is able to reliably transmit energy while transmitting command information in text and other complicated formats. On this basis, the microsystem can not only monitor the surrounding temperature independently over the long term but also dynamically adjust its temperature thresholds according to the received commands to implement the abnormal temperature alarm more properly. Overall, owing to the increase in the energy transfer rate and information transfer rate, US‐TENG‐MF‐SWIPT significantly facilitates the autonomy and interaction capability of various intelligent microsystems.

## 4 Experimental Section

### Measurement

The experiment employs a digital storage oscilloscope (DSOX4024A, Keysight) for signal measurement. A simple multifrequency ultrasonic generator, assembled from multiple fixed‐frequency signal generators, a relay group, and several multifrequency ultrasonic probes, was used to transmit energy and information to the TENG. A digital multimeter (FLUKE 17B/MAX) was used to measure detailed parameters, such as the open‐circuit voltage output from the TENG. The upper‐level computer software, written in Visual Basic language, was used to configure and send the information.

### Experimental System

First, the TENG was placed 10 mm below the ultrasonic probe. Then, an ultrasonic coupling agent was applied to fill the area between the TENG and the ultrasonic probe, completely covering the probe and the upper surface of the TENG. The two electrodes of the TENG were connected to the input ports of the receiving circuit, whereas the oscilloscope was connected to the voltage detection port of the energy storage capacitor and the information demodulation port. The ultrasound probe was simultaneously connected to multiple fixed‐frequency signal generators, with the output of the corresponding ultrasound frequencies controlled through a relay group and the designed computer software.

### Experimental procedure

The ultrasound probe was initially connected to a 25 kHz signal generator to charge the energy storage capacitor. Once the oscilloscope observes that the voltage across the energy storage capacitor no longer increases significantly, the upper‐level computer software sends the information. The transmitting circuit's microprocessor receives information via the RS‐422 bus. The microprocessor subsequently generates a modulation signal, which controls the opening and closing of the relay group output contacts through an optocoupler and Metal Oxide Semiconductor Field Effect Transistor, thereby controlling the connection of the corresponding frequency signal generator to the ultrasound probe to produce ultrasound at different frequencies. The ultrasound generated by the ultrasound probe then acts on the TENG, and the output signal from the TENG was processed by the receiving circuit's information demodulation module, generating voltage signals with varying amplitudes. These were recognized by the receiving STM32L0 low‐power microcontroller as corresponding information (00/01/10/11). Finally, the STM32L0 microcontroller uses the acquired information and energy to control the LCD display and temperature sensor. After the preset temperature threshold was displayed, the current temperature was displayed at regular intervals. If the current temperature exceeds the preset threshold, the alarm light turns red.

## Conflict of Interest

The authors declare no conflict of interest.

## Supporting information



Supporting Information

Supporting Information

## Data Availability

The data that support the findings of this study are available from the corresponding author upon reasonable request.
