# Supplementary material for: Simultaneous Multifrequency Modulated Wireless Information and Power Transfer for a Triboelectronic Monitoring System
Source: Adv Sci (Weinh). 2025 Sep 28;12(45):e10427. doi: 10.1002/advs.202510427 (PMC12677592; doi:10.1002/advs.202510427)
Supplement: Supplementary file 1 — Supporting Information [file ADVS-12-e10427-s001.docx]

Supporting Information

Simultaneous Multifrequency Modulated Wireless Information and Power Transfer for a Triboelectronic Monitoring System

Hongwei Yuan^#^, Youngwook Chung^#^, Ze Wang, Haojie Li, He Zhang, Sang-Woo Kim*, Keren Dai*

**Note S1.** Optimization problem model of the SWIPT system.

The optimization model mentioned in the text is as follows,

$\begin{matrix} \text{ }\min_{\left\{R1,R2,R3,C1,C2\right\}} \text{ }\sum_{i=1}^{x} P_{ed}^{i} \\ \text{ s.t. min}\left\{\Delta U1,\text{ }\Delta U2,\text{ }\ldots,\text{ }\Delta U_{N-1}\right\}>\text{ }V_{dmin} \\ \text{ max}\{\Delta U1,\text{ }\Delta U2,\text{ }\ldots,\text{ }\Delta U_{N-1}\}<V_{s}/\left(N-1\right) \\ \text{ }t_{dch}\text{ }<\alpha t_{d}\text{ and }t_{ch}\text{ }<\beta t_{d} \\ \text{ }t_{ch}\text{ }>\gamma\cdot1/f_{\text{min}}\text{ and }t_{dch}\text{ }>5\times1/2f_{\text{max}} \\ \text{ }U_{N}<V\text{ and }U_{0}>0 \end{matrix}$ (S1)

It can be seen that there are many parameters in the optimization model and the constraints are complex. Therefore, the optimization problem is solved in a distributed manner in combination with the combination characteristics of the circuit. The information demodulation circuit consists of two parts: a high-pass filter circuit and a rectifier filter circuit. The high-pass filter circuit realizes the regulation of the signals’ amplitude of different frequencies, and the rectifier filter circuit rectifies the sinusoidal signal into an approximate DC signal, thereby simplifying the optimization problem to:

（1）High-pass filter circuit optimization problem

$\begin{matrix} \text{ }\min_{\left\{R1,C1\right\}} \text{ }\sum_{i=1}^{x} P_{ed}^{i} \\ \text{ s.t. min}\left\{\Delta U1,\text{ }\Delta U2,\text{ }\ldots,\text{ }\Delta U_{N-1}\right\}>\text{ }V_{dmin} \\ \text{ max}\{\Delta U1,\text{ }\Delta U2,\text{ }\ldots,\text{ }\Delta U_{N-1}\}<V_{s}/\left(N-1\right) \\ \text{ }U_{N}<V\text{ and }U_{0}>0 \end{matrix}$ (S2)

（2）Rectification and filtering circuit optimization problem

$\begin{matrix} \text{ }\min_{\left\{R2,R3,C2\right\}} \text{ }\sum_{i=1}^{x} P_{ed}^{i} \\ \text{ s.t. }t_{dch}\text{ }<\alpha t_{d}\text{ and }t_{ch}\text{ }<\beta t_{d} \\ \text{ }t_{ch}\text{ }>\gamma\cdot1/f_{\text{min}}\text{ and }t_{dch}\text{ }>5\times1/2f_{\text{max}} \end{matrix}$ (S2)

For high-pass filter circuits, in order to reliably identify information, first, the cutoff frequency *f*_0_ in the amplitude-frequency curve of the high-pass filter circuit needs to be set to the center value of the ultrasonic frequency, and the product of the resistor R1 and the capacitor C1 can be obtained; Then, the impedance-frequency characteristics of the multi-layer ceramic capacitor (MLCC) are used to select the capacitance range; and further find the optimal values of the resistor and capacitor with the help of heuristic algorithm. The process is shown in Figure. S1.

For the rectifier filter circuit, combined with $t_{ch}=R_{2}C_{2}$ and $t_{dch}=\left(R_{2}+R_{3}\right)C_{2}$，In order to reduce power consumption and simplify calculation, set R3 to 2*R2, and the value range of can be obtained from the constraint function.

The resistance and capacitance range of commonly used manufacturers (such as ROHM and YAGEO) are adopted, and the resistance and capacitance of 0603 packages are selected. The maximum resistance of ROHM company is 10MΩ, and the minimum capacitance of YAGEO company is 1 pF. The calculated results are shown in Supplementary Figure. S4. In the figure, the horizontal and vertical axes are the index corresponding to 10, and the intersection of the red and blue parts is the parameter value range that satisfies the condition. It can be seen that the larger the resistance, the smaller the capacitance, the larger the load impedance after the diode, and the higher of the *U_out_*. The value of 5.1MΩ, 0.047nF capacitor in the lower right corner of the condition is selected for simulation using LTspice software.

**Note S2.** The bit error rate (BER) analysis of the SWIPT system.

We conduct a study of BER performance of the proposed system over an additive white Gaussian noise (AWGN) across a wide range of SNR values (-10~20 dB).

1. **Theoretical Analysis of System BER**

The BER of the proposed information‐loading scheme can be evaluated by referring to the conventional four-level pulse amplitude modulation (PAM4) calculation method. Specifically, the information symbols “11,” “10,” “01,” and “00” correspond to voltage levels of 0.33, 1.01, 1.78, and 2.58 V, respectively, with each symbol lasting for 0.2 s. The received signal is modeled as the transmitted signal corrupted by additive Gaussian white noise with a standard deviation of δ. For symbol demodulation, the decision thresholds are set at the midpoints between adjacent voltage levels.

$$V_{th1}=\frac{0.33+1.01}{2}, V_{th2}=\frac{1.01+1.78}{2}, V_{th3}=\frac{1.78+2.58}{2}$$

Assuming equal probabilities for all symbol occurrences, the error probability can be derived using the Gaussian tail function$Q(x)=\frac{1}{\sqrt{2\pi}}\int_{x}^{\infty} e^{-t^{2}/2}\text{d}t$, and the average BER is expressed in Equation (S4).

$\begin{matrix} P_{e1 }=Q(\frac{V_{th1} -0.33}{\sigma} ), \\ P_{e2 }=Q(\frac{1.01 -V_{th1}}{\sigma} )+Q(\frac{V_{th2} -1.01}{\sigma} ), \\ P_{e3 }=Q(\frac{1.78-V_{th2}}{\sigma} )+Q(\frac{V_{th3} -1.78}{\sigma} ), \\ P_{e4 }=Q(\frac{2.58-V_{th3}}{\sigma} ), \end{matrix}$ (S4)

$P_{s}=\frac{P_{e1}+P_{e2}+P_{e3}+P_{e4}}{4}.$ (S5)

Considering that each symbol carries 2 bits and adopting Gray coding, the BER is further reduced, as described in Equation (S6). This theoretical analysis provides a reference for both simulation and experimental validation.

$P_{s}^{bit}\approx\frac{P_{s}}{2}$ (S6)

1. **Simulation Analysis of System BER**

In the simulations, 5,000 symbols were generated, each carrying 2 bits, with the SNR varied from –10 to 20 dB. The corresponding results are shown in Figure S7, where the BER is observed to decrease progressively as the SNR increases.

The BER of the proposed system decreases rapidly with increasing SNR, approaching the theoretical performance in AWGN channels. At low SNR, symbol errors are more frequent due to noise-induced misidentification, whereas at higher SNR the received symbols match the ideal levels. These results demonstrate that the information loading method reliably transmits multiple bits per symbol with high fidelity under varying noise conditions.

**Note S3.** The calculation process of the system’s operational lifetime under continuous monitoring conditions

After completing the energy-information transmission in the experiment, the storage capacitor was charged to 4.2 V using 125 kHz ultrasound, subsequently powering the buck LDO, microcontroller, and temperature sensor.

1. Energy calculation of the storage capacitor

A low-dropout regulator (LDO) with low power consumption and compact size, TPS7A0222PDBVR, manufactured by Texas Instruments (TI), was selected. (<https://www.ti.com.cn/cn/lit/ds>/symlink/tps7a02.pdf ?ts=1755701693949&ref_url=https%253A%252F%252Fwww.ti.com.cn%252Fzh-cn%252Fpower-management%252Flinear-regulators-ldo%252Fproducts.html) with an output of 2.2 V. When the capacitor voltage dropped to 2.250V, the LDO could no longer maintain a stable 2.2 V output. In the experiment, the storage capacitor had a capacitance of 11 mF, corresponding to a stored charge of 2.145 × 10⁻^2^ C according to Equation (S7).

$Q=C\cdot\Delta V$ (S7)

1. Load Power Consumption

The microcontroller used in the system is an STM32L0 (https://www.st.com.cn/zh/microcontrollers-microprocessors/stm32l011d3.html), which operates in two modes: Stop mode without RTC when no information is received, and low power run mode during information reception. The supply voltage of the microcontroller is 2.2 V. In low power run mode, the current is 7 μA, corresponding to a power consumption of **15.4 μW**; In Stop mode without RTC, the current is 0.29 μA, corresponding to a power consumption of **0.638 μW**.

The temperature sensor selected was the MS1088 from MICRODUL (https://microdul.com/en/assets/public/images/content/MS1088D_Datasheet_M90-32-0665.pdf) .The LDO TPS7A02, manufactured by Texas Instruments (TI), exhibits a quiescent current (Iq) of 25 nA.

Accordingly, the load current in sleep and active modes was calculated, as summarized in Table S1

**Table S1.** Load Current in sleep and active Modes

| Part Number | Current in active ode  (uA) | Current in sleep mode  (uA) |
| --- | --- | --- |
| STM32L0 | 7 | 0.29 |
| MS1088 | 75 | 0.02 |
| TPS7A0222PDBVR | 0.025 | 0.025 |
| **Total Current**  **(uA)** | **82.025** | **0.335** |

During periodic environmental temperature measurements without processor intervention, the charge consumption per cycle was calculated as follows: in sleep mode, 60 min consumed 1.206 × 10⁻^3^ C; in active mode, the MS1088 sensor requires 50 ms for a single temperature conversion; And with an I²C communication frequency of 80 kHz and a total data length of 47 bits, the overall duration for one measurement and data acquisition is approximately 50.5875 ms. Using the corresponding Equation (S8), the charge consumption in active mode was 4.048 × 10⁻⁶ C.

$Q=\left(I_{out}+I_{q}\right)\cdot t$ (S8)

Based on these values, the device operating time under different temperature measurement intervals was calculated, as summarized below,

**Table S2**. Calculation of System Operating Time

| Temperature Measurement Interval (min) | Single-Cycle Charge of sleep mode(×10^-3^C) | Number of Work Cycles | Operating Time  （hour） |
| --- | --- | --- | --- |
| 60 | 1.210 | 17 | 17 |

It can be seen that completing a single energy–information transmission is sufficient to sustain device operation for **17 hours**.

**Note S4.** The comparative discussions with existing commercial wireless solutions

The proposed system was compared with four commonly used low-power commercial wireless communication modules: STMicroelectronics’ STM32WB, Texas Instruments’ CC2530, Nordic Semiconductor’s nRF24L01+, and Espressif’s ESP32-C61. These modules support Bluetooth, ZigBee, and 2.4 GHz Wi-Fi 6 applications. Their power consumption is summarized in Table S3.

Specifically:

1. **STM32WB**: The minimum current is 1.576 milliampere, and with the minimum operating voltage of 1.7 volt, the resulting power consumption is 2.679 milliwatt. (https://www.st.com/en/microcontrollers-microprocessors/stm32wb05kz.html)
2. **CC2530**: The minimum current is 5.3 milliampere, and with the minimum operating voltage of 1.71 volt, the resulting power consumption is 9.063 milliwatt. (https://www.ti.com/lit/ds/symlink/cc2340r2.pdf?ts=1754843186576)
3. **nRF24L01+**: In the receive mode, the minimum current is 12.6 milliampere at 250 kbps, and with the minimum operating voltage of 1.9 volt, the resulting power consumption is 23.94 milliwatt. (https://cdn.sparkfun.com/assets/3/d/8/5/1/-nRF24L01P_Product_Specification_1_0.pdf)
4. **ESP32-C61**: In the receive mode, the minimum current is 81 milliampere, and with the minimum operating voltage of 3 volt, the resulting power consumption is 243 milliwatt. (https://www.espressif.com.cn/sites/default/files/documentation/esp32-c61_datasheet_en.pdf)

The results indicate that the minimum power consumption of current commercial modules is 2.679 milliwatt, nearly 20 times higher than the 140.31 mW of the proposed system. Such high power demand limits compatibility with the output capabilities of TENGs, rendering these commercial modules unsuitable for scenarios requiring simultaneous energy harvesting and information transfer. Moreover, the system enables SWIPT, allowing continuous energy collection even during the information transfer process. In terms of size, the complete system has been realized in a 20 mm × 20 mm footprint, well matched to the dimensions of the TENG device. These features collectively highlight the suitability of the proposed design for miniaturized, self‐powered sensing applications where low power consumption, multifunctionality, and small size are critical.

**Table S3.** The electrical parameters of the commercial wireless modules

| Part number | Manufacture | Voltage  (V) | Current  (μA) | Power  (μW) | Connectivity supported |
| --- | --- | --- | --- | --- | --- |
| stm32wb | STMicroelectronics | 1.7 | 1,576 | 2,679 | Bluetooth® LE and 2.4 GHz radio solution |
| cc2340 | TEXAS INSTRUMENTS | 1.71 | 5,300 | 9,063 | 2.4-GHz IEEE 802.15.4 and ZigBee |
| nRF24L01+ | Nordic Semiconductor | 1.9 | 12,600 | 23,940 | Bluetooth Thread Zigbee 802.15.4 2.4 GHz |
| ESP32-C61 | Espressif | 3 | 81,000 | 243,000 | 2.4 GHz Wi-Fi 6 + Bluetooth 5 (LE) + Thread/Zigbee SoC |

1. **STM32WB.** The minimum current is 1.576 mA, and with the minimum operating voltage of 1.7 V, the resulting power consumption is **2.679 mW**. (<https://www.st.com/en/microcontrollers-microprocessors/stm32wb05kz.html>)
2. **cc2530.** The minimum current is 5.3 mA, and with the minimum operating voltage of 1.71 V, the resulting power consumption is **9.063 mW**. (https://www.ti.com/lit/ds/symlink/cc2340r2.pdf?ts=1754843186576)
3. **nRF24L01+**. In the receive mode, the minimum current is 12.6 mA at 250 kbps, and with the minimum operating voltage of 1.9 V, the resulting power consumption is **23.94 mW**. (https://cdn.sparkfun.com/assets/3/d/8/5/1/nRF24L01P_Product_Specification_1_0.pdf)
4. **ESP32-C61.** In the receive mode, the minimum current is 81 mA, and with the minimum operating voltage of 3 V, the resulting power consumption is **243 mW**.

(https://www.espressif.com.cn/sites/default/files/documentation/esp32-c61_datasheet_en.pdf)

**Figure S1.** Optimization method for high-pass filter module parameter values.


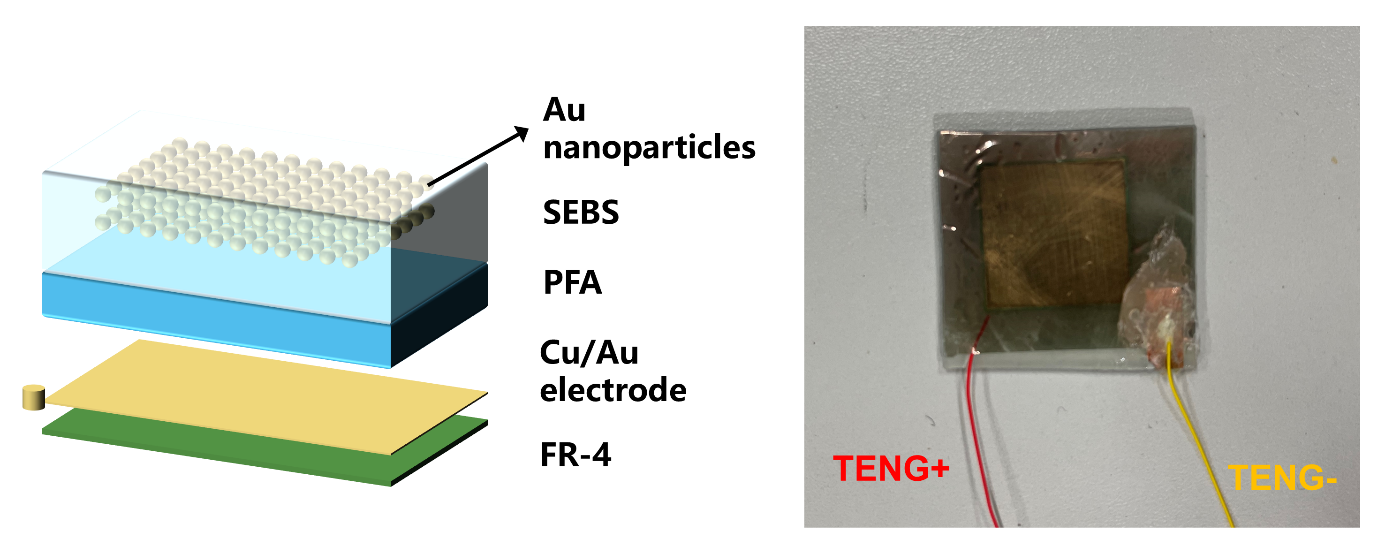


**Figure S2.** Exploded view and photograph of the US-TENG . The US-TENG combined a thin (~25 μm) perfluoroalkoxy (PFA) membrane with a flexible electrode that contains SEBS layers confining Au nanoparticles in the middle.


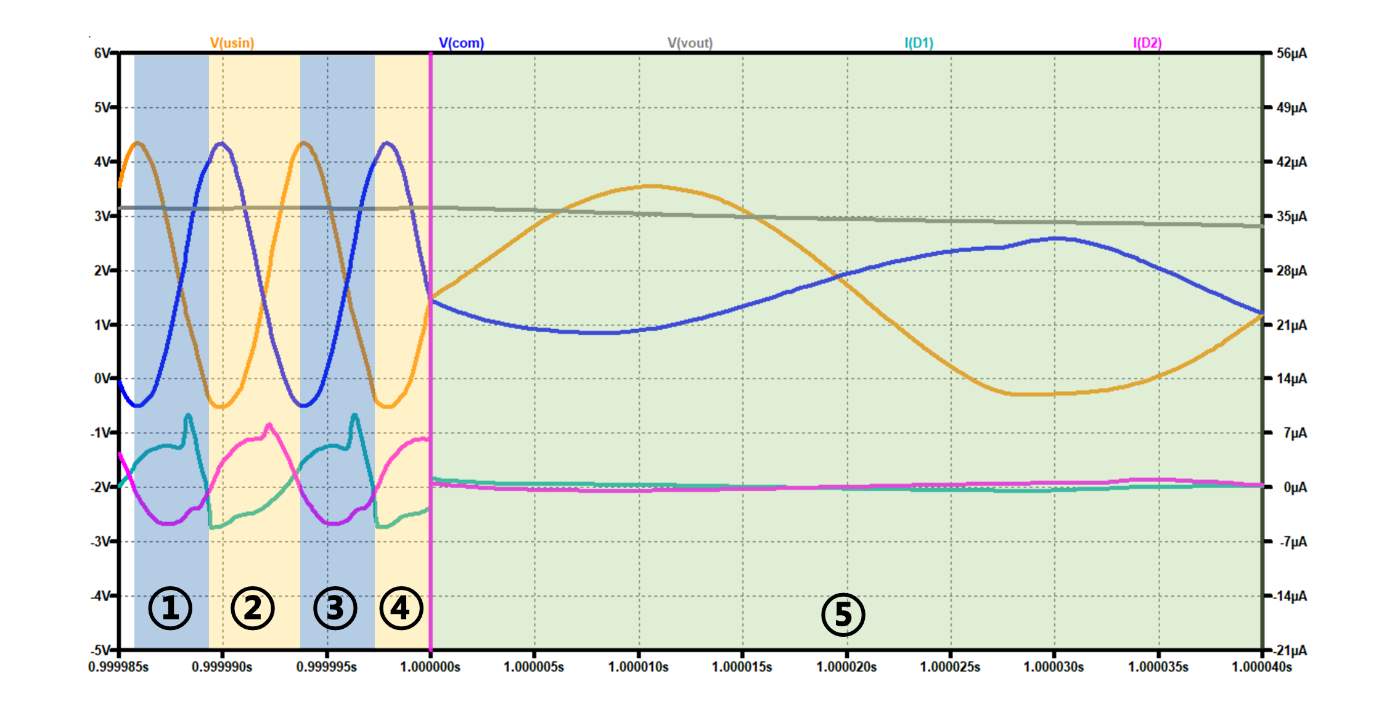


**Figure S3.** Working process diagram of the proposed information demodulation circuit


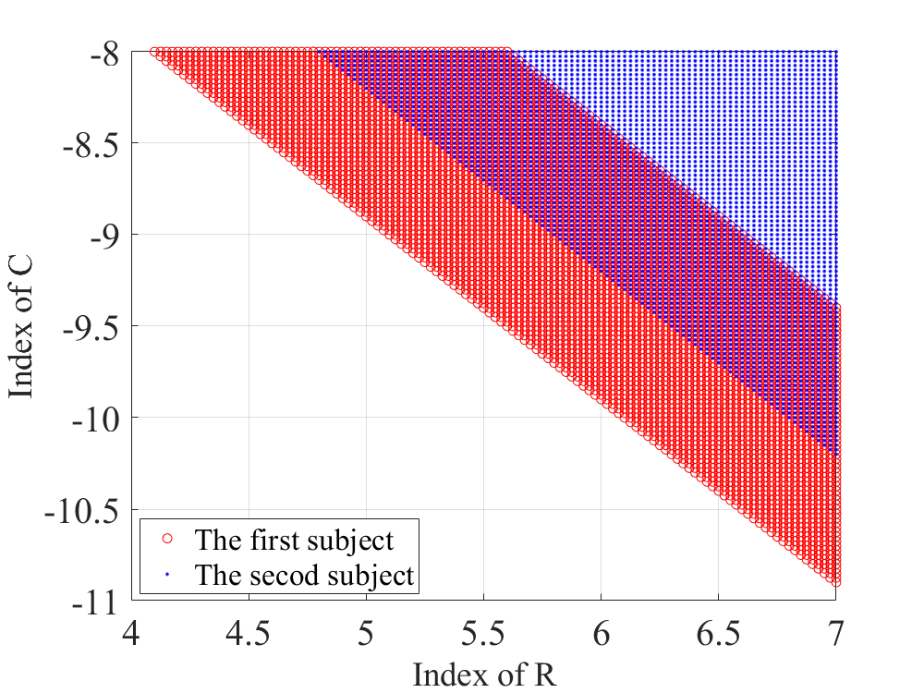


**Figure S4.** Simulation results of parameter range of traditional circuit.


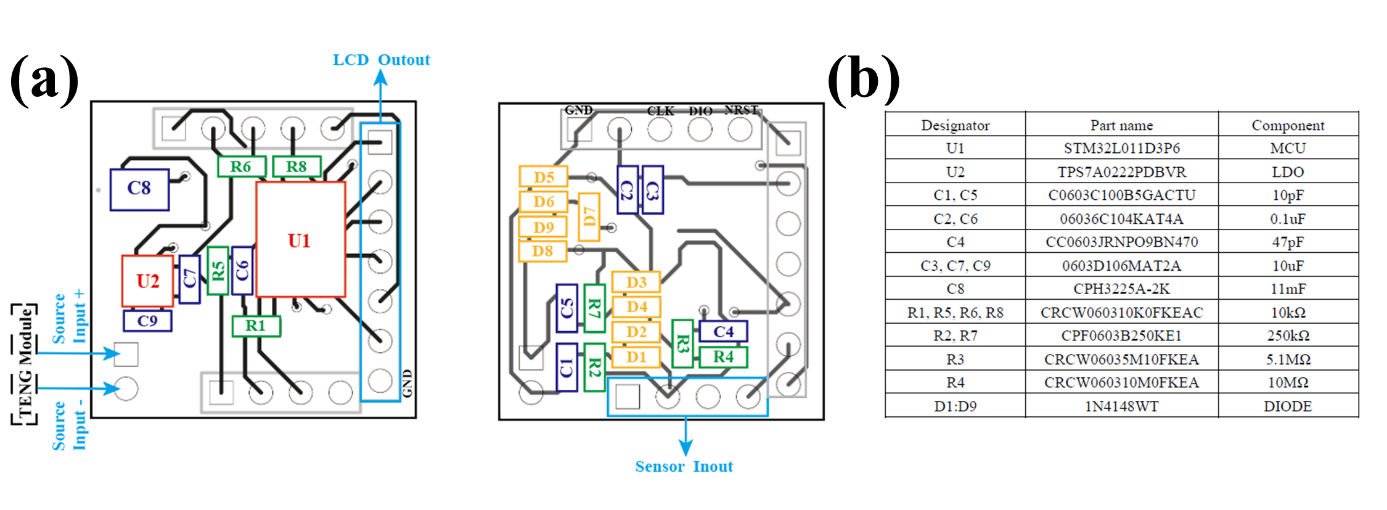


**Figure S5.** Layout design and components of electronic circuit module for multi-frequency information demodulation and simultaneous power transmission system. PCB layout design for electronic circuit. (a) The top layer and the bottom layer of the electronic circuit. The black lines represent the metal layer, and the circuit components are categorised with different colours. The input pins receives the power source and information generated by the TENG module and the output pins display LCD screen according to the received information. (b) The table showing the details of the components.


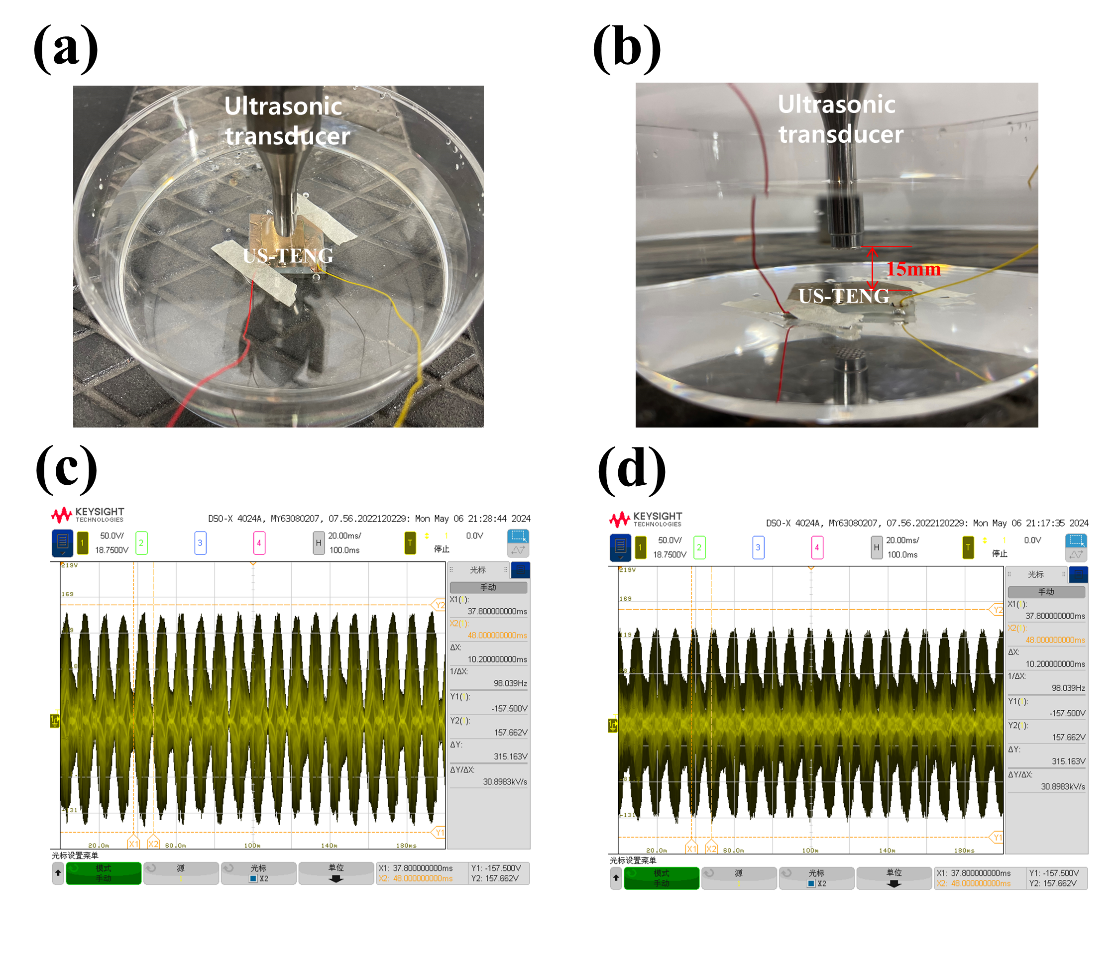


**Figure S6.** Underwater output of the US-TENG (a,b) Underwater measurement setup (c) Voltage output at 125KHz (d) Voltage output at 25KHz


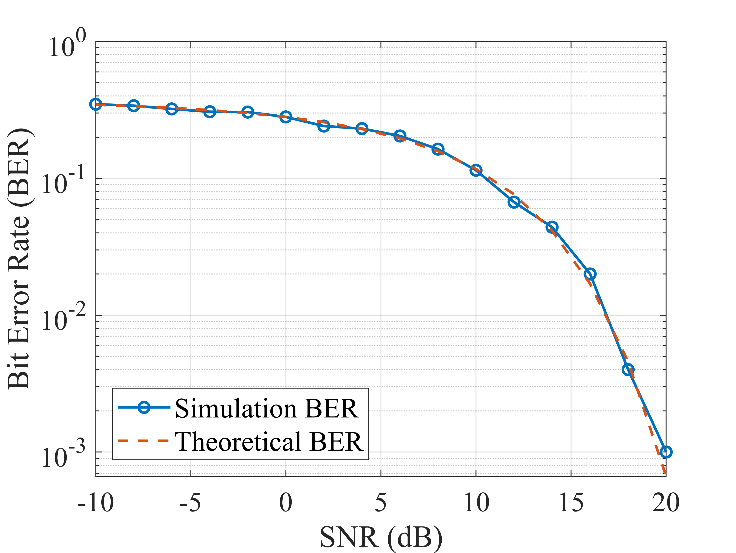


**Figure S7.** BER of the system under AWGN channel


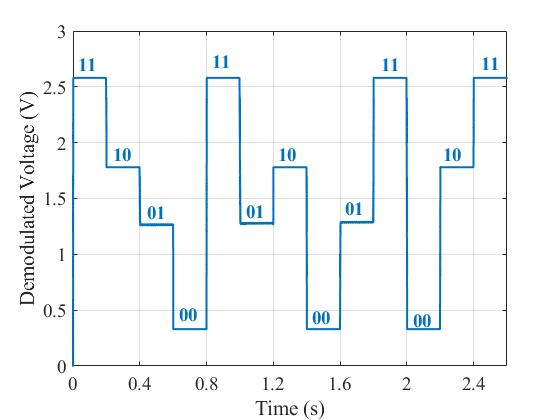


**Figure S8.** Simulation results of multi-group data transmission capability
